# Co-Circulation of Phleboviruses and *Leishmania* Parasites in Sand Flies from a Single Site in Italy Monitored between 2017 and 2020

**DOI:** 10.3390/v13081660

**Published:** 2021-08-21

**Authors:** Mattia Calzolari, Giuseppe Romeo, Emanuele Callegari, Paolo Bonilauri, Chiara Chiapponi, Elena Carra, Gianluca Rugna, Roberta Taddei, Davide Lelli, Michele Dottori

**Affiliations:** Istituto Zooprofilattico Sperimentale della Lombardia e dell’Emilia Romagna (IZSLER) “B. Ubertini”, Via Bianchi, 9, 25124 Brescia, Italy; giuseppe.romeo@izsler.it (G.R.); emanuele.callegari@izsler.it (E.C.); paolo.bonilauri@izsler.it (P.B.); chiara.chiapponi@izsler.it (C.C.); elena.carra@izsler.it (E.C.); gianluca.rugna@izsler.it (G.R.); roberta.taddei@izsler.it (R.T.); davide.lelli@izsler.it (D.L.); michele.dottori@izsler.it (M.D.)

**Keywords:** sand fly, *phlebotomus perfiliewi*, *phlebotomus perniciosus*, *leishmania infantum*, *phlebovirus*, toscana virus, fermo virus, corfou virus, ponticelli I virus, ponticelli II virus, ponticelli III virus

## Abstract

Sand flies transmit *Leishmania infantum*, which is responsible for causing leishmaniasis, as well as many phleboviruses, including the human pathogenic Toscana virus. We screened sand flies collected from a single site between 2017 and 2020 for the presence of both phleboviruses and *Leishmania*. The sand flies were sampled with attractive carbon dioxide traps and CDC light traps between May and October. We collected more than 50,000 sand flies; 2826 were identified at the species level as *Phlebotomus perfiliewi* (98%) or *Phlebotomus perniciosus* (2%). A total of 16,789 sand flies were tested in 355 pools, and phleboviruses were found in 61 pools (6 Toscana virus positive pools, 2 Corfou virus positive pools, 42 Fermo virus positive pools, and 7 Ponticelli virus positive pools, and 4 unidentified phlebovirus positive pools). *Leishmania* was found in 75 pools and both microorganisms were detected in 16 pools. We isolated nine phleboviruses from another 2960 sand flies (five Ponticelli viruses and for Fermo viruses), not tested for *Leishmania*; the complete genome of a Fermo virus isolate was sequenced. The simultaneous detection in space and time of the Fermo virus and *L. infantum* is evidence that supports the co-circulation of both microorganisms in the same location and partial overlap of their cycles. A detailed characterization of the epidemiology of these microorganisms will support measures to limit their transmission.

## 1. Introduction

Phleboviruses (family: *Phenuiviridae,* genus: *Phlebovirus*) are ssRNA viruses characterized by a tri-segmented genome. The L segment (6.4 kb) codes the RNA-dependent RNA polymerase (RdRp), the M segment (3.2 kb) encodes for a polyprotein that is the origin of several proteins, and the S segment (1.7 kb) encodes for two proteins with an ambisense strategy (https://viralzone.expasy.org/252 accessed on 10 May 2021). Species in the genus were previously defined by the degree of serological cross-reactivity, but the frequent isolation of new viruses and the reassorting capacities of these viruses suggested a need to change the classification standards [1]. More than 60 phleboviral species have now been defined using a threshold of 95% identity in the amino acid sequences of the RdRp [2].

Some sand fly-borne phleboviruses cause disease in humans. One example, Toscana virus (TOSV), is the etiological agent of neuroinvasive diseases including meningitis and meningoencephalitis. The TOSV was first isolated in 1971 [3] and later recognized as an agent of meningitis in humans [4]. This virus has been detected in large parts of many Mediterranean countries, from Spain to Turkey [5]. Previously unknown phleboviruses have been detected in sand flies, but little is known about their pathogenic potential, ecological cycles, mechanisms of persistence, or presence in vertebrate reservoirs.

Several of these viruses had been detected in Northern Italy. In addition to TOSV, three phleboviruses—Ponticelli I virus, Ponticelli II virus, and Ponticelli III virus—were isolated [6]. These three viruses differ in their M segments, likely as a result of reassortant events. In the same area, Fermo and Corfou viruses, in addition to phleboviruses possessing sequences with no known among previously described strains, were found in sand flies [7].

Among the various sand fly-borne pathogens, the intracellular protozoa of the *Leishmania* genus (family: *Trypanosomatidae*; order: *Kinetoplastida*) contribute significantly to human diseases in Italy and elsewhere. The species circulating in the Mediterranean region include *L. donovani* and *L. infantum*, which cause visceral leishmaniasis (VL), and *L. tropica* and *L. major*, which, together with dermotropic *L. infantum* strains, cause cutaneous leishmaniasis (CL). The estimated annual incidence of leishmaniasis in this area is 1200–2000 cases of VL and 239,500–393,600 cases of CL [8].

*Leishmania infantum* is almost the only causal agent of leishmaniasis in Italy, where it causes VL and CL in humans, as well as canine leishmaniasis (canL) in dogs. Dogs are regarded as the main source of *Leishmania* infection in many epidemiological settings, but other domestic and wild animal species have also been implicated as reservoirs [9].

VL and CL are endemic to the hilly part of Emilia-Romagna. The first outbreak of 60 cases of VL was recorded in 1971–1972 [10], and a recrudescence of human leishmaniasis was reported to have occurred in 2012–2013, with more than 30 cases of VL [11,12]. Recent epidemiological studies based on molecular typing suggest that dogs cannot be the main reservoir for human leishmaniasis in this area because the strains involved in VL cases are only distantly related to canine *Leishmania* strains compared to other strains [13,14].

Phleboviruses and *Leishmania* share the same principal vectors: sand flies. Sand flies are tiny hematophagous insects that often live close to domestic animals and humans. Only females bite, typically from dusk to dawn. They are poor fliers, flying silently for short distances, and they have activity peaks during summertime in non-tropical regions; sand flies are widely distributed in Mediterranean countries. The two species that are most abundant in the natural and rural environments of the Emilia-Romagna region are *Ph. perniciosus* and *Ph. perfiliewi*; both species are proven vectors of *Leishmania infantum* and TOSV in Italy [15].

In this work, we report the contemporaneous detection of diverse phleboviruses and *Leishmania* parasites in sand flies collected at a site in the Valsamoggia municipality (BO) in the Emilia-Romagna region, Italy from 2017 to 2020.

## 2. Materials and Methods

### 2.1. Sampled Site

Between 2017 and 2020, we sampled a single site that had already been surveyed, beginning in 2012 (44°17′15″ N, 11°41′46″ E) [16]. This site was located in the hilly area of the Emilia-Romagna region (altitude 196 m) in the garden of an uninhabited building. The surroundings were characterized by a typical hilly landscape with cultivated fields and hedges interspersed by plots of woodlands, which were prevalent on the hilltops.

### 2.2. Sand Fly Sampling and Identification

Sampling was performed fortnightly at night in the period from May to October. We used two models of traps: traps baited with dry ice, to produce carbon dioxide (CO_2_), and standard miniature CDC light traps. One carbon dioxide trap was used at the same site for every sampling and was used as an estimate of the abundance of sand flies. Other traps were employed in variable numbers, from one to nine, to maximize the number of insects collected, keeping a minimum distance of 20 m between them to avoid reciprocal interference (Table S1). A subset of the captured sand flies was clarified in chlorolactophenol and morphologically identified using a light microscope using specific morphological keys [17,18]. We grouped other sub-samples of sand flies in pools, which were submitted to biomolecular analysis for phlebovirus and *Leishmania* detection or utilized for virus isolation in cell cultures.

Weather data from the closest climatic stations were retrieved with the web app of the Regional Environmental Protection Agency (www.arpae.it accessed on 10 April 2021), Dext3r (https://simc.arpae.it/dext3r/ accessed on 10 April 2021). The average of the precipitation recorded at four stations (Guiglia, Monte San Pietro, Bazzano, Savignano sul Panaro) was used, and average temperatures were recorded at the Guiglia station.

### 2.3. Pathogen Detection and Sequencing

We examined pools of 50 females for biomolecular analysis. The pools were ground, and the genetic material was extracted with an automated extractor (BioSprint 96, Qiagen, Germany) and retro-transcribed. We searched phleboviruses with PCR (henceforth pan-phlebo-PCR), targeting a 370-nucleotide region of the S segment [19]. The amplicons obtained with the pan-phlebo-PCR were sequenced, and the sequences were used to identify detected viruses by BLAST analysis in the GenBank database (https://blast.ncbi.nlm.nih.gov/Blast.cgi accessed on 10 April 2021). We detected *Leishmania* parasites with real-time PCR targeting the kinetoplast DNA [20]. The minimum infection rates (MIR) of *Leishmania* were calculated for every sample, assuming the presence of at least one *Leishmania*-positive sand fly per positive pool. Primers of the PCRs are reported in Table S2.

A Fermo virus isolate was sequenced on a MiSeq Instrument (Illumina Inc., San Diego, CA, USA) as described elsewhere [6]. The sequences obtained were aligned with homologous sequences that were available in Gen Bank using MAFFT [21], and they were used to obtain a neighbor-joining tree and maximum likelihood trees with the IQ-Tree software [22].

### 2.4. Virus Isolation

A sub-sample of the females was grouped in 25-specimen pools on a surface kept at −80 °C, and tested for isolation as quickly as possible, as reported previously [6]. The insects were ground in a glass potter in minimal essential medium supplemented with penicillin and streptomycin and were centrifugated at 3000× *g* for 15 min. An aliquot of these homogenates was submitted to pan-phlebo-PCR. The samples were inoculated in a confluent monolayer of VERO cells (African green monkey kidney cells) at passage 170 (cell culture collection of IZSLER, code BSCL86) and incubated at 37 °C with 5% CO_2_. We observed the cultures daily for 7 days to detect and characterize cytopathic effect (CPE). Then, we sub-cultured the cryolysates twice into fresh monolayers. Pools that were utilized for virus isolation were not tested for the presence of *Leishmania*.

## 3. Results

### 3.1. Sampled Sand Flies

**Figure 1:**
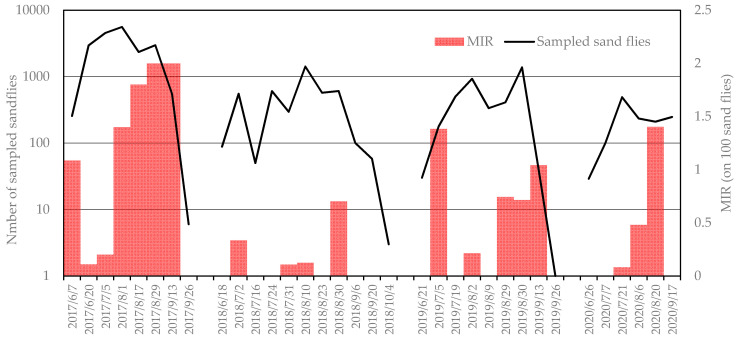
Number of sand flies sampled per night per trap (logarithmic scale) with reference to the *Leishmania* minimum infection rates per 100 specimens.

**Figure 2:**
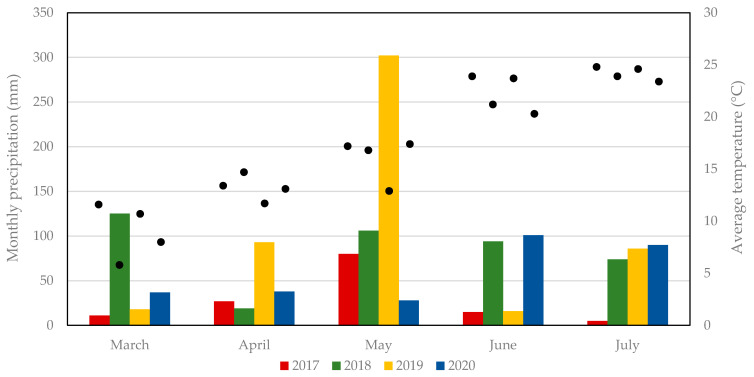
Monthly precipitation (histograms) and average temperature (black circles) from March to July at the sampled sites during the period of surveillance.

We collected 50,510 sand flies during the study (2017–2020), and morphologically identified 2826 specimens: 2622 were identified as *Ph. perfiliewi* (98%) and 45 were identified as *Ph. perniciosus* (2%) (Table 1). The number of sand flies collected in CO_2_ traps revealed the great abundance of sand flies in 2017 compared to the other years (Figure 1), with a record of 5600 insects in one trap in August. The averages of the temperatures and monthly precipitation recorded in the climatic stations near the sampling site are reported in Figure S1. Interestingly, the 2017 season, which was characterized by a large number of sandflies, had a drier spring than the other years; the lowest precipitation was between March and June (Figure 2, Table S3).

### 3.2. Molecular Analyses

**Figure 3:**
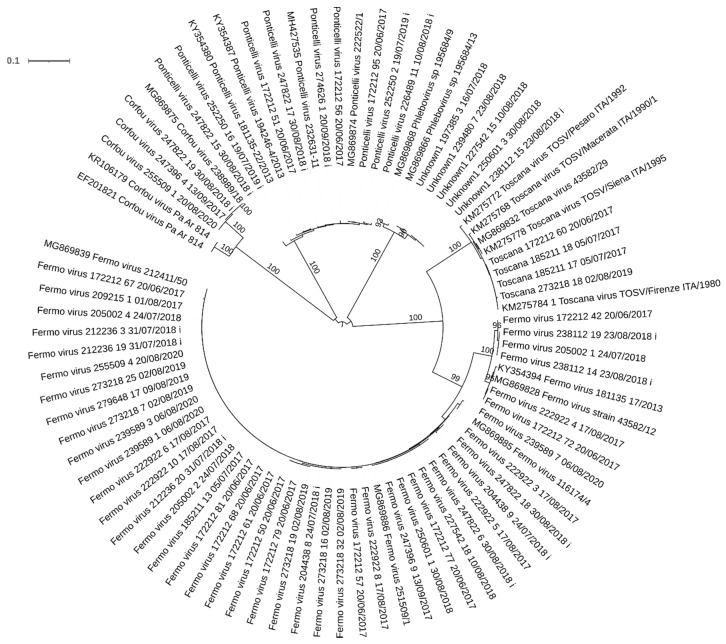
Neighbor joining tree of partial sequences of the S segments obtained from the field samples with reference to the strain collection day and utilization for isolation (i); the selected homologous sequences are available in GenBank (by referencing the accession number), bootstrap supports >90% showed near the branch.

We tested 16,789 sandflies (Table 1)—grouped in 355 pools—using PCR. Of these, 75 tested positive for *Leishmania* through the PCR, and 61 produced a sequence ascribable to a phlebovirus: 6 Toscana viruses, 42 Fermo viruses, 7 Ponticelli viruses, 2 Corfou viruses, and 4 of a putative phlebovirus that had been detected in the surveyed area (Table 2). The partial sequence of the putative phlebovirus was distantly related to other phleboviruses detected in Mediterranean countries, but it showed the highest amino acid identity with the South American phleboviruses Anhanga virus (67.5% GB: NC_033837). The neighbor joining tree obtained with these sequences and the previously deposited sequence of the same virus are shown in Figure 3 (Jukes–Cantor model, bootstrap resampling of 1000). Both *Leishmania* and phleboviruses were detected in 16 pools (14 Fermo viruses, 1 Ponticelli virus, 1 Corfou virus) (Table 2). Using only data concerning the more detected virus, the Fermo virus, on the 34 days of sampling, we detected both Fermo virus and *Leishmania* parasite in 15 days, while we detect only Fermo virus in 2 days and only *Leishmania* in 3 days. Considering the days of sampling, the detection of both Fermo virus and *Leishmania* was significantly related, with an odds ratio of 8 (95% confidence, 1.74–36.71, *p* < 0.01).

The MIRs of *Leishmania* recorded in different seasons are shown in Figure 1. The 2017 season, which was characterized by a great abundance of sand flies, showed more intense *Leishmania* circulation (Figure 1), and the proportion of *Leishmania*-positive pools in 2017 differed significantly from the proportion in the other years (42/110 in 2017 vs. 37/255 in 2018–2020, Pearson χ^2^, *p* < 0.001).

### 3.3. Virus Isolation

**Figure 4:**
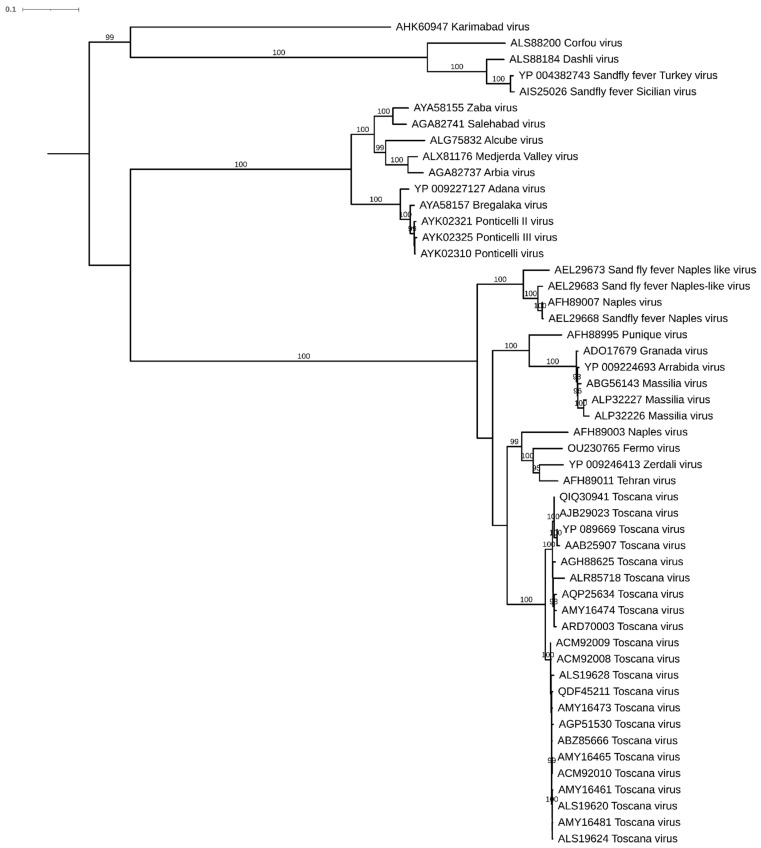
Maximum likelihood tree obtained using the amino acid sequences of the RpRd of complete sequences of the Fermo virus and homologous sequences of sand fly-borne phleboviruses detected in Mediterranean and Middle Eastern countries. GenBank accession numbers reported as reference, bootstrap supports >90% showed near the branch.

We attempted to isolate virus from 120 pools—100 in 2018 (collected from 18 June to 20 September) and 20 in 2019 (collected on 19 July)—for a total of 2960 sand flies (Table 1). We detected phleboviruses in 20 of these pools (10 Fermo viruses, 7 Ponticelli viruses, 1 Corfou virus, and 1 virus referred to as Unknown1). Cytopathic effects—from moderate to evident—were observed in nine cultures, which were all sampled in 2018. Virus isolation was then confirmed by testing the cryolysates by pan-phlebo PCR, confirming the isolation of four strains of Fermo virus and five strains of Ponticelli virus (Table 3). Interestingly, one of these Ponticelli virus strains was isolated from a homogenate that tested negative in the pan-phlebo PCR and another that was isolated from a homogenate in which a Fermo virus sequence was detected. We submitted the isolated Fermo virus (strain 212236-3) for next generation sequencing and obtained the complete genome, which was then deposited in ENA (European Nucleotide Archive) with the accession numbers OU230765 (L segment), OU230766 (M segment) OU230767 (S segment), to make available the first complete genome sequence of that virus. The amino acid RdRp sequence of this virus showed an identity of 92.1% with the closest deposited virus, the Tehran virus (GB JF939846), which was isolated from *Ph. perfiliewi* in Iran in 1976. The maximum likelihood tree obtained with the Fermo RdRp amino acid sequence is shown in Figure 4 (LG + F + R3 model selected by IQ-Tree software, ultrafast bootstrap approximation of 1000 iterations). We were able to confirm a high identity between the complete genome nucleotide sequences and the partial Fermo virus sequences deposited in GenBank, which was ascribable to the L segments (between 92.1% and 95.2%) or the S segments (between 91.7% and 99.7%). High identity was also recorded with respect to partial Balkan virus sequences detected in sand flies in Balkan countries in 2015 [23]. More precisely, an identity of between 86.9% and 89.1% was found for the partial L segments, and an identity of from 88.5% to 89.5% was found for the partial S segments.

## 4. Discussion

Sand flies were abundant at this study site, as demonstrated by the 5600 specimens collected in one trap in one night in 2017. This abundance was mainly sustained by the *Ph. perfiliewi* species, which was much more abundant than *Ph. perniciosus* at the same site, as previously recorded in the neighboring area [16,24]. The abundance of sand flies differed by year, and we hypothesize that this was influenced by weather conditions; in particular, there was a correlation between abundance and low precipitation in the spring months. A similar relation has been shown for *Lutzomyia* species in South America [25,26]. As already noted in this area of Italy, the abundance of sand flies directly affected the circulation of Toscana virus [27]. Similarly, the recrudescence of human *Leishmaniasis* was recorded in the Emilia-Romagna region after seasons with abundant captures of sand flies, such as in 2013–2014 [6,28]. Interestingly, a relationship between drought conditions and the 1970–1971 outbreak was already hypothesized by Pampiglione [10]. If this relationship can be precisely characterized, the definition of factors that influence the number of sand flies will also help in assessing the intensity of pathogen circulation and may, perhaps, be useful in predicting increases of infections.

We recorded the presence of at least four different phleboviruses at the same site during the four seasons of surveillance, similarly to the results obtained in the geographical area around the site [7]. In addition to the well-known TOSV, we confirm the presence of Corfou virus, which was first isolated in Greece in 1981 [29], and of Fermo virus, which was first isolated in the Marche region of Italy in 2012 [30], about 250 km from our site. In addition, amplicons referable to Ponticelli virus were generated, although we did not type them further, as more information on the M segment sequence was needed. We also sequenced amplicons that had already been generated from other sites (defined as Unknown1) and that were ascribable to a putative phlebovirus that has not been isolated; this phlebovirus has been detected since 2013 as far as about 80 km from this site in Emilia-Romagna [6]. The detection of these viruses through a non-virus species-specific PCR, with an intrinsic limit on the sensitivity, suggests that they had a greater presence. Among them, TOSV has the greatest relevance to health because of its capacity to cause neuroinvasive infections. The other phleboviruses detected have not been clearly associated with diseases in humans, though serological reactions to Ponticelli II virus were observed in sick persons in the neighboring Lombardia region [31]. Interestingly, only with this virus have serological reactions been detected, while the other two reassortant viruses—which had different M segments (Ponticelli I virus and Ponticelli III virus)—did not show any reactivity. This highlights the importance of the M segment in defining the ability of these viruses to infect vertebrates, and the ability to reassort this segment is likely an important factor in determining the evolution and pathogenicity of phleboviruses, similar to that observed with viruses in the *Orthobunyavirus* genus [32,33]. The close proximity of these phleboviruses detected at the same site likely offers them the opportunity to reassort among themselves.

The isolates obtained confirmed the major adaptability of the Ponticelli viruses to cell cultures, highlighting their ability to displace other viruses that are present in an insect pool, as observed with the isolation of one Ponticelli virus from a pool that was positive for Fermo virus. Fermo virus also caused a weak CPE, suggesting a minor adaptability to cell culture; despite this, we were able to isolate and make available the complete genome of Fermo virus. Its relationship with the closest Tehran virus confirmed that Fermo virus represents a new phlebovirus species according to the 95% threshold in the RdRp sequence. The virus is closely related to Balkan viruses; however, in the absence of a complete sequence of the L segment, it is not possible to draw firm conclusions on their relation. The possible presence of a vertebrate host of this virus and its potential pathogenicity require additional studies.

Leishmaniasis is a well-known human health threat, and the surveyed site is located in an area in which the recrudescence of VL caused by *L. infantum* has been recorded since 2012 [11,28]. Moreover, a screening of blood donors performed in 2014–2015 using serological methods resulted in detection of asymptomatic infections in 12.5% of the tested individuals, indicating an important cumulative exposure to the parasite in the same area [34]. The high rate of infection recorded in this study attests to the active circulation of these parasites in sand flies. However, the reasons for the recent upsurge in VL cases in Emilia-Romagna are unknown. The seroprevalence of *L*. *infantum* in the canine population in the Emilia-Romagna region is low when compared to the CanL seroprevalence in other endemic areas of Italy, and it has shown a decreasing trend over time [13]. It is noteworthy that previous molecular studies showed the circulation of two genetically distinct populations of *L. infantum* in the Emilia-Romagna region—one affecting dogs and the other circulating in *Ph. perfiliewi* and humans [13,14,16]. This suggests the existence of two overlapping transmission cycles that probably involve different reservoirs.

The co-circulation of *L. infantum* and phleboviruses has already been described in the Mediterranean basin [35,36,37,38]; we confirmed this phenomenon by detecting the contemporary presence of both in the surveyed sites. We were also able to assess the correlation between the most commonly detected phlebovirus (Fermo virus) and *Leishmania*, and we found a significant relationship in the co-detection of both on particular days of sampling. The simultaneous detection in space and time of the Fermo virus and *Leishmania* is an evidence of the cycle similarity between the two microorganisms and strongly suggests an overlap between their cycles. An obvious point of convergence was the sharing of the vector, *Ph. perfiliewi*, which was the most abundant sand fly species recorded and the main vector of *L. infantum* in this area [16]. Interestingly, this species was also incriminated as one of the main vectors for TOSV. It will be of interest to determine whether these two microorganisms share other epidemiological characteristics, such as reservoirs. *Leishmania* sampled in that area showed a unique epidemiology, which strongly suggests the presence of a reservoir that has not yet been determined. Additional studies are required to determine if this statement can be extended to other protozoa and Fermo viruses. The possibility of its similarity to the ecology of phleboviruses can help in clarifying the cycles of both.

## 5. Conclusions

Sand flies can host a variety of microorganisms, including viruses and parasites, in strict spatial and temporal coexistence. The definition of the cycles of these microorganisms and the possible interactions between them could lead to better understanding of this complex picture. The potential ability to increase the infective capacity of *Leishmania* through coinfection with phleboviruses also has been suggested [39]. The cycles of sand fly-transmitted pathogens are often enigmatic because it can involve different species of sand fly and different reservoirs in different areas. The advances in the cycle characterization of one of these microorganisms can help in clarifying the cycle of another. An important experimental effort must be made to define the cycle of these microorganisms, which can lead to the assessment of the risks linked to their presence and development of control measures necessary for limiting their transmission.

## Figures and Tables

**Table 1:** Collection, testing, and identification of sand flies during the years of surveillance.

		2017	2018	2019	2020	Total
Collected sand flies		25,966	7696	12,559	4289	50,510
Identified	*Ph. perfiliewi*	768	1123	427	453	2771
	*Ph perniciosus*	7	22	13	13	55
Tested	*Leishmania*/Pan-phlebo PCR	5443	3363	5893	2090	16,789
	Isolation in cell cultures		2460	500		2960

**Table 2:** *Leishmania* spp. and phleboviruses detected in double-tested pools by year of surveillance. Phlebovirus-positive pools that also tested positive for *Leishmania* spp. are shown in parentheses.

	2017	2018	2019	2020	Total
Tested pools	110	73	126	46	355
*Leishmania*	42	5	22	6	75
Toscana virus	4		2		6
Fermo virus	23 (10)	5 (1)	8 (2)	6 (1)	42 (14)
Ponticelli virus	4	2 (1)	1		7 (1)
Corfou virus	1 (1)			1	2 (1)
Unknown1		4			4

**Table 3:** Results of the analysis of the sand fly pools (25 specimens) with successful isolation.

Data	Pool Code	Homogenate PCR	CPE ^1^	Cryolysate PCR
24/07/2018	204438-8	Fermo virus	++	Fermo virus
24/07/2018	204438-9	Fermo virus	+	Fermo virus
31/07/2018	212236-3	Fermo virus	++	Fermo virus
31/07/2018	212236-20	Fermo virus	+	Fermo virus
10/08/2018	226489-11	Ponticelli virus	++	Ponticelli virus
30/08/2018	247822-15	Ponticelli virus	++	Ponticelli virus
30/08/2018	247822-17	Ponticelli virus	++	Ponticelli virus
30/08/2018	247822-18	Fermo virus	++	Ponticelli virus
06/09/2018	256303-2	Neg.	++	Ponticelli virus

^1^ Cytopathic effect.

## Data Availability

Fermo virus sequences are available at ENA (https://www.ebi.ac.uk/ena/browser/home accessed on 10 April 2021), other data are available on request from the corresponding author.

## Supplementary Materials

The following are available online at https://www.mdpi.com/article/10.3390/v13081660/s1: Figure S1: Average temperature (running mean of 15 days) and monthly precipitation in the sampled site during the period of surveillance; Table S1: Sampled, tested, and identified sand flies for every day of sampling; Table S2: PCR primers used in this work; Table S3: Details of the average temperature and monthly precipitation recorded during the survey.
