# Supplementary material for: Co-Circulation of Phleboviruses and Leishmania Parasites in Sand Flies from a Single Site in Italy Monitored between 2017 and 2020
Source: Viruses. 2021 Aug 21;13(8):1660. doi: 10.3390/v13081660 (PMC8402820; doi:10.3390/v13081660)
Supplement: Supplementary file 1 [file viruses-13-01660-s001.zip › viruses-1287891-supplementary.pdf]

Table S1: Sampled, tested, and identified sand flies for each day of sampling.

Day of sampling	CO_2 traps	CDC light traps	Tested	*Ph. perfiliewi*	*Ph. perniciosus*	Total
07/06/2017	1		184	72		256
20/06/2017	1		2750	200	2	2952
05/07/2017	1		4450	98		4548
01/08/2017	1		5500	96	4	5600
17/08/2017	2		4598	102		4700
29/08/2017	2		6700	99	1	6800
13/09/2017	2		1000	98		1098
26/09/2017	2		9	3		12
18/06/2018	1		54	35		89
02/07/2018	1		452	100		552
16/07/2018	1	1	68	20		88
24/07/2018	2	1	830	209	9	1048
31/07/2018	2	3	1872	240	8	2120
06/08/2018		1	2			2
08/08/2018		1	1			1
10/08/2018	1		1319	99	1	1419
13/08/2018		1	1			1
23/08/2018	1	2	749	177	2	928
30/08/2018	1	2	882	167	2	1051
06/09/2018	2	1	263	60		323
20/09/2018	1	1	58	9		67
04/10/2018	1	1		7		7
07/06/2019		2	0			0
21/06/2019	1	2	24	11	2	37
05/07/2019	1	1	346	92	4	442
19/07/2019	2	3	1410	100		1510
02/08/2019	1	8	6144	183	3	6330
09/08/2019	1	1	393			393
29/08/2019	1	9	1930			1930
30/08/2019	1	1	1680			1680
13/09/2019	1	3	191	41	4	236
26/09/2019	1	1	1			1
26/06/2020	1	2	229			229
07/07/2020	1	1	233	60	4	297
21/07/2020	1		2305			2305
06/08/2020	1	1	416	118	3	537
20/08/2020	1	1	479	189	6	674
03/09/2020	1		0			0
17/09/2020	1		161	86		247
Total			47684	2771	55	50510

Table S2: PCR primers used in this work

	Primers	Sequence	Ref.
Pan-Phlebo-PCR	Phlebo f1	TTTGCTTATCAAGGATTTGATGC	[19]
	Phlebo f2	TTTGCTTATCAAGGATTTGACC	
	Phlebo r	TCAATCAGTCCAGCAAAGCTGGGATGCA TCAT	
Leishmania real-time PCR	leish-F	ACTTTTCTGGTCCTCCGGGTAG	[20]
	leish-R	CCTATTTTACACCAACCCCCAGT	
	leish Pb	ATTTCTGCACCCATTTT	

Table S3: Details of the average temperature and monthly precipitation recorded during the survey.

	2017		2018		2019		2020	
	t (°C)	Prec (mm)	t (°C)	Prec (mm)	t (°C)	Prec (mm)	t (°C)	Prec (mm)
January	0.9	3	5.6	14	3.6	40	5.8	23
Febrary	5.9	69	1.7	188	7.0	31	9.0	1
March	11.6	11	5.8	125	10.7	18	8.0	37
April	13.4	27	14.7	19	11.7	93	13.1	38
May	17.2	80	16.8	106	12.9	302	17.4	28
June	23.9	15	21.2	94	23.7	16	20.3	101
July	24.8	5	23.9	74	24.6	86	23.4	90
August	26.0	14	24.4	58	24.4	13	24.4	50
September	17.3	92	20.4	17	19.2	47	19.6	51
October	15.1	6	15.2	59	15.8	49	13.1	73
November	7.8	213	9.0	73	9.3	258	9.2	32
December	4.5	43	4.9	25	6.3	49	4.5	

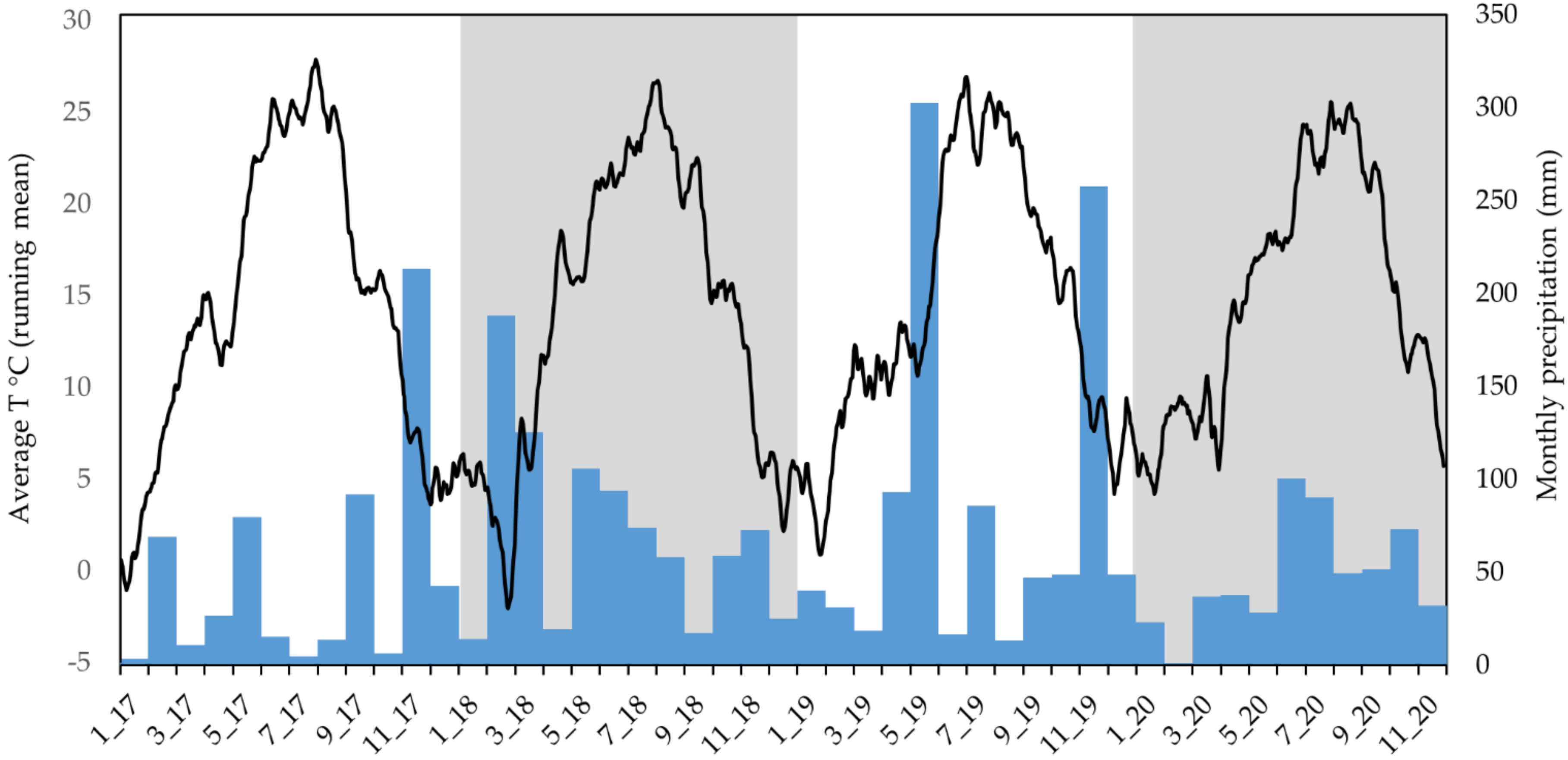

Figure S1: Average temperature (running mean of 15 days) and monthly precipitation at the sampled site during the period of surveillance.
